# Preventive Behaviors during the COVID-19 Pandemic in Delaware’s Underserved Communities: A Longitudinal Study (2021–2022)

**DOI:** 10.3390/bs14100890

**Published:** 2024-10-01

**Authors:** Sharron Xuanren Wang, Nicole Bell Rogers, Kylie Trask, Dorothy Dillard, Melissa A. Harrington

**Affiliations:** 1 Department of Sociology and Criminal Justice, Delaware State University, Dover, DE 19901, USA; 2 Department of Nursing, Delaware State University, Dover, DE 19901, USA; nbell@desu.edu; 3 Department of Neuroscience, Duke University, Durham, NC 27708, USA; kat82@duke.edu; 4 Center for Neighborhood Revitalization and Research, Delaware State University, Dover, DE 19901, USA; ddillard@desu.edu; 5 Department of Biology, Delaware State University, Dover, DE 19901, USA; mharrington@desu.edu

**Keywords:** COVID-19, preventive behaviors, underserved communities, racial disparities, health disparities

## Abstract

The COVID-19 pandemic officially started in March 2020 and ended in May 2023. Preventive behaviors have been proven to be one of the most effective strategies for preventing COVID-19 transmission. Common preventive behaviors against COVID-19 include but are not limited to wearing a face mask, washing and sanitizing one’s hands frequently, avoiding crowds, and avoiding traveling. Underserved communities and racial/ethnic minorities across the U.S. have been disproportionately affected by the COVID-19 pandemic. Using a three-wave longitudinal survey conducted from early 2021 to late 2022, the present study investigated changes in the adoption of COVID-19-preventive behaviors among residents living in Delaware’s most underserved communities. We also examined whether changes in COVID-19-preventive behaviors differed by race. Our results indicated that fewer people adopted preventive behaviors as the pandemic progressed, and this finding is applicable to both Black people and White people. However, Black people had a consistently greater likelihood of adopting preventive behaviors compared to White people from early 2021 to late 2022. Scientific and policy implications are discussed.

## 1. Introduction and Background

On 11 March 2020, the World Health Organization (WHO) declared COVID-19 a pandemic. About three years later, on 5 May 2023, the WHO announced that COVID-19 was no longer a global health emergency. In response to the announcement, the U.S. federal Public Health Emergency (PHE) for COVID-19 ended on 11 May 2023. The COVID-19 pandemic had a significant negative impact in terms of individual health, finances, and social wellbeing. However, a number of measures have proven effective at preventing the spread of COVID-19, including regular testing, vaccination, and preventive behaviors (e.g., mask-wearing, hand sanitization, social distancing, etc.). Testing has been important for identifying, tracing, and treating COVID-19 cases while also helping to inform critical policy decisions, such as whether to reopen businesses or restrict public gatherings. Vaccines have been a critical tool in decreasing the number of new infections, minimizing the likelihood of severe infections, and slowing the pandemic (CDC, 2021). However, individual access to testing and vaccination may differ depending on structural or individual-level factors [1,2,3,4]. For example, testing kits and vaccines may have been unevenly distributed across neighborhoods [4], with underserved neighborhoods receiving less aid [2]. (Underserved neighborhoods or underserved communities usually have limited access to resources, including healthcare resources. Disadvantaged populations typically reside in underserved neighborhoods or communities, including racial/ethnic minorities, people living in rural areas, and people who are disadvantaged in terms of socioeconomic status, among other residents). In addition, many studies have shown that African Americans are less likely to be vaccinated against COVID-19 than White people [3,5]. For example, a Kaiser Health News report revealed that Black Americans were vaccinated at rates two to three times lower than those of White Americans [6]. The lower rate of vaccination among African Americans might be attributed to structural factors (i.e., lower access to primary care and limited availability of vaccines in minority neighborhoods) as well as individual factors (i.e., mistrust toward the medical system). COVID-19 vaccine hesitancy may be high among African Americans. For example, a report using data from December 2020 suggested that 49% of Black Americans expressed COVID-19 vaccine hesitancy, compared to 34% of White people [7]. Another study conducted in Delaware’s underserved communities also found that Black residents had higher vaccine hesitancy compared to their White counterparts [3].

While testing and vaccination have been instrumental in preventing the spread of the disease, their effectiveness has been largely dependent on their availability. Preventive behaviors, on the other hand, are readily available strategies anyone can use to decrease the likelihood of infection and transmission of the virus. Adopting preventive practices has been especially crucial in preventing the spread of the virus among people who may be resistant to COVID-19 testing and vaccination, including residents in underserved communities. Several preventive practices (summarized in Table 1) have proven to be effective. These behaviors include but are not limited to frequently washing and sanitizing hands, avoiding travel, and wearing a mask. Studies have indicated that multiple factors, including social, cultural, economic, personal, and educational factors can affect one’s preventive behavior uptake [8]. In addition, public attitudes about these behaviors may vary across individuals and time. For example, whether to require that people wear a mask in public spaces has been a contentious issue [9]. In addition, having limited access to certain resources (i.e., masks, sanitizer, etc.) or being an essential worker may limit the types of preventive measures a person can uptake.

Previous studies have identified distinct socioeconomic and demographic factors that affect the use of preventive behaviors against COVID-19. For self-protective behaviors such as social distancing, handwashing, and mask-wearing, a nationwide study conducted during the early stages of COVID-19 in April 2020 revealed that older individuals and those living with vulnerable individuals were more likely to adopt all types of preventive behaviors and maintain these behaviors even after the mandates were lifted. In contrast, there was a lower probability of adopting preventive behaviors among people living with children under 18 years of age [10]. Moreover, socioeconomic status also predicted preventive behavior uptake. Specifically, low-income individuals were less likely to increase preventive behaviors compared to higher-income individuals [10]. In addition, the US COVID-19 Trends and Impact Survey reported a geographic difference in preventive behavior, whereby people in the Northeast and West Regions of the U.S. demonstrated fewer contacts and greater mask use compared to individuals in the South and Midwest Regions [11]. Researchers also observed increased protective behaviors at the community level depending on the rising and falling of COVID-19 cases and whether a person had been in close contact with the virus [12]. In terms of racial differences in preventive behavior uptake, Roberts and David (2021) found that the African Americans in their study sample reported higher levels of preventive behaviors and self-efficacy than Caucasians [13]. Similarly, in another cross-sectional study, researchers found that Black people were more likely to wear face masks than White people [14].

Another factor that might affect people’s uptake of preventive behaviors may be their fear of contracting COVID-19. Ahorsu and colleagues (2022) suggested that fear is critical in defining the characteristics of an infectious disease [15]. Fear of a disease can be positively associated with its transmission, morbidity, and mortality rate. If an individual does not feel that they are susceptible to contracting the virus or if they do not feel that the disease represents a severe threat, they are less likely to fear the disease and engage in the recommended preventive behaviors.

Underserved communities across the nation have suffered from having limited resources to combat COVID-19 [16]. Health disparities have worsened during the pandemic. For example, counties with a higher proportion of Black residents and adverse social determinants of health had significantly higher COVID-19 mortality rates [17]. In addition, several reports have indicated that Black and Hispanic Americans have higher mortality rates related to COVID-19 than their White counterparts [18,19]. Although the COVID-19 public health emergency has officially ended, data collected during the pandemic can help us better understand the social determinants of infection so that we may be better equipped for future health emergencies. This line of research is especially important for understanding the needs of underserved communities during pandemics and epidemics. Using data from a three-wave longitudinal survey collected from Delaware’s underserved communities, the present study examines changes in COVID-19-preventive behaviors from early 2021 to the end of 2022, and whether changes in preventive measures differ by race. In addition, factors associated with preventive behavior adoption will be identified.

## 2. Methods

### 2.1. Recruitment

Participants were recruited from the most underserved communities across Delaware’s three counties (New Castle County, Kent County, and Sussex County) from early 2021 to late 2022. Nine communities (three from each county) were selected based on their Community Health Index (CHI) scores. Delaware’s Division of Public Health (DE DPH) uses the Community Health Index (CHI) as a common indicator for characterizing community health at the census tract level. The CHI is a composite score derived from several measures, including life expectancy, infant mortality rate, percentage of high school graduates, and child poverty rate. The communities where we conducted the present study ranked low in terms of these community health measures. Moreover, as expected, these communities demonstrate high poverty rates, limited health resources, and large minority populations. For recruitment in these communities, we collaborated with two trusted community health advocacy agencies: the Wilmington Hope Commission (WHC) and the Sussex County Health Coalition (SCHC). The WHC and SCHC currently serve as two of the key coordinating organizations in carrying out Delaware’s COVID-19 response.

The first wave of data was collected between 4 March 2021 and 30 October 2021; the second wave of the survey was conducted from 16 September 2021 to 25 May 2021; and the third wave was conducted from 31 March 2021 to 11 November 2021. Two hundred and fifty-three participants completed all 3 surveys (N = 253). Using listwise deletion for missing data, 208 participants remained in the dataset. All study participants were 18 years of age or older and resided in Delaware’s most underserved communities when participating in the study. The first survey included questions about demographic and socioeconomic characteristics, COVID-19-related practices, and general health. Questions regarding COVID-19-related preventive behaviors were adopted from the COVID-19 Community Response Survey developed by John Hopkins University. The 2 follow-up surveys only contained time-variant questions, including questions about COVID-19-related practices and general health. Surveys were conducted electronically through REDCap. REDCap is a HIPAA-compliant web-based application for developing and managing surveys and databases. It is widely used by researchers in Public Health, Epidemiology, and other fields. Participants used iPads provided by the study team to complete the surveys at the study sites (nurses, student interns, staff, and participants wore masks at all times, sanitized their hands and iPads frequently, and remained 6 feet apart). Based on the population characteristics in the communities, surveys were made available in English, Spanish, and Haitian Creole.

### 2.2. Measures

The outcome variables of interest are a series of COVID-19-related preventive behaviors. These behaviors include more handwashing than usual (yes = 1/no = 0); more use of hand sanitizer than usual (yes = 1/no = 0); more cleaning your home than usual (yes = 1/no = 0); more disinfecting surfaces in household than usual (yes = 1/no = 0); disinfecting or wiping down groceries (yes = 1/no = 0); disinfecting or wiping down mail or packages (yes = 1/no = 0); stocking up on food and supplies; avoiding or canceling domestic traveling (yes = 1/no = 0); avoiding or canceling international traveling (yes = 1/no = 0); not ordering takeout from restaurants (yes = 1/no = 0); and wearing a mask when out in public (yes = 1/no = 0).

### 2.3. Covariates

The key independent variable in the analysis is racial/ethnic background. Race/ethnicity includes non-Hispanic White people, non-Hispanic Black people (“Non-Hispanic Black”, “NH-Black”, and “African American” are used interchangeably), and Others. These categories are mutually exclusive. Others include Hispanics, Native Americans, and Asians. Due to their small sample size, we grouped them into one category (Others). Several demographic and social characteristics served as covariates. Sex includes female (Yes = 1) and male (Yes = 0). The socioeconomic variables included educational level (lower than high school = 1; high school and higher = 0), health insurance status (private health insurance, public health insurance, and no insurance), annual family income lower than USD 20,000 (yes = 1), and Fear of COVID-19 scale (FCV-19S) score.

The FCV-19S is a validated mental health questionnaire that measures an individual’s COVID-19-related fears [20]. The FCV-19S includes seven items, and participants are instructed to rate their level of agreement with each statement. The seven items are (1) “I am most afraid of corona”; (2) “It makes me uncomfortable thinking about corona”; (3) “My hands become clammy when I think about corona”; (4) “I am afraid of losing my life because of corona”; (5) “When I watch news and stories about corona on social media, I become nervous or anxious”; (6) “I cannot sleep because I’m worrying about getting corona”; and (7) “My heart races or palpitates when I think about getting corona”. The respondents rated their level of agreement with each statement using a 5-point Likert scale, including “strongly disagree”, “disagree”, “neutral”, “agree”, and “strongly agree”. The FCV-19S ranges from 7 to 35 and is calculated by adding up the responses to each item (i.e., 1–5). A higher score indicates a greater fear of COVID-19.

It should be noted that certain demographic covariates, such as race/ethnicity, sex, and educational level, only appeared in the first baseline survey. Health insurance status, family income, and the FCV-19S were assessed across the 3 waves of the survey. Additionally, we tested for interactions, including interactions between preventive behaviors and race; however, our analysis demonstrated no significant interactions.

## 3. Results

Table 2 displays the descriptive statistics. In our longitudinal sample, 58% of the respondents were female. In terms of racial and ethnic background, 36.06% identified as non-Hispanic Black (NH-Black), 47.6% identified as non-Hispanic White (NH-White), and 16.35% identified as “Other”. In terms of educational attainment, approximately 20% of the respondents reported that they had less than a high school diploma. Regarding the time-variant characteristics, in Wave 1, 35% of the respondents reported that their previous year’s household annual income was lower than USD 20,000. This percentage was 37% in Wave 2 and 34% in Wave 3. In addition, the majority of the respondents had public health insurance: 57.21% in Wave 1, 60.58% in Wave 2, and 59.13% in Wave 3. Interestingly, the percentage of respondents who did not have health insurance increased from 11.54% to 16.35% from Wave 1 to Wave 3. Unsurprisingly, the fear of COVID-19 (based on the FCV-19S) decreased from Wave 1 to Wave 3 (15.94 to 13.14), indicating that the sample decreased its fear of COVID-19 over time. Finally, approximately 59% of the respondents had received at least one dose of the COVID-19 vaccine in Wave 1, and this number increased to 70% in Wave 3.

In terms of the outcome variables of interest (i.e., COVID-19-related preventive behaviors), two trends emerged in the descriptive results: (1) the number of preventive behaviors in Wave 1 was high, and (2) compared to Wave 1, the number of preventive behaviors in Wave 3 decreased dramatically. The results suggested that, at the beginning of the COVID-19 pandemic, most people were cautious and adopted preventive behaviors. However, as time progressed, people were not as proactive in carrying out preventive behaviors, as observed in Wave 3. For example, in Wave 1, 92% of the respondents reported yes to “More handwashing than usual”. This number decreased to 73% in Wave 3. In total, 91% percent of the respondents reported yes to “more hand sanitizer than usual” in Wave 1, and this number decreased to 70% in Wave 3. In total, 81% reported yes to “cleaning your home more than usual”, and this number decreased to 58% in Wave 3. In total, 90%reported yes to “More disinfecting surfaces in your household than usual”, and this figure decreased to 63% in Wave 3. In total, 69% reported yes to “disinfecting or wiping down groceries”, and this number decreased to 44% in Wave 3. In total, 60% reported yes to “disinfecting or wiping down mail or packages”, and this figure decreased to 38% in Wave 3. In total, 79% reported yes to “Stocking up food and supplies”, and this figure decreased to 53% in Wave 3. In total, 72% reported yes to “Avoiding or canceling domestic travel”, and this figure decreased to 42% in Wave 3. In total, 68% of respondents reported yes to “avoiding or canceling international travel”, and this percentage decreased to 43% in Wave 3. In total, 45% reported yes to “Not ordering takeout from restaurants”, and this figure decreased to 27% in Wave 3. Finally, 90% reported yes to “Wearing a mask when out in public”; this number decreased to 57% in Wave 3.

Table 3 displays descriptive statistics by race and by wave. The results suggested that across the three waves, there was a greater percentage of NH-Black people with annual incomes below USD 20,000 than NH-White people. In addition, Black people and Others demonstrated higher fear of COVID-19 than White people. Furthermore, across the three waves, NH-Black people had the lowest percentage of vaccinations against COVID-19 among the groups.

In terms of COVID-19-preventive behaviors by race, two interesting trends emerged: (1) across the 11 preventive behaviors we examined, NH-Black people reported a greater percentage of preventive behaviors than NH-White people across all three waves; (2) the percentage of preventive behaviors decreased dramatically among NH-White people from Wave 1 to Wave 3, whereas the percentage among NH-Black people only showed a moderate decrease.

Table 4 displays the results from the logistic regression models predicting the odds ratio of COVID-19-preventive behavior adoption in Wave 1. Among all the preventive behaviors we tested, 4 behaviors differed significantly by race in Wave 1. For example, NH-Black individuals had an odds ratio of 2.58 (*p* = 0.006) for “disinfecting or wiping down mail or packages”. The results indicated that the odds of NH-Black individuals disinfecting or wiping down mail or packages were 1.6 times greater than the odds of NH-White people when controlling for all the covariates. In addition, the odds of NH-Black individuals stocking food and supplies were almost twice as high as the odds of NH-White individuals when controlling for all the covariates (OR = 2.92, *p* = 0.015). The odds of NH-Black individuals to avoid or cancel international travel were 1.7 times greater than the odds for NH-White individuals when controlling for all the covariates (OR = 2.66, *p* = 0.008). Finally, the odds of NH-Black individuals not ordering takeout from restaurants were 1.3 times greater than the odds of NH-White individuals when controlling for all the covariates (OR = 2.33, *p* = 0.011).

Table 5 and Table 6 display the results from the logistic regression models predicting the odds ratios of COVID-19-preventive behavior adoption in Wave 2 and Wave 3, respectively. Interestingly, the odds ratios for all preventive behaviors were statistically significant for NH-Black people in both Wave 2 and Wave 3. In addition, all the odds ratios for NH-Black people were greater than 1, indicating that NH-Black people had greater odds of enacting all the tested preventive behaviors compared to NH-White people (i.e., more handwashing than usual, more hand sanitizer than usual, more cleaning the home than usual, more disinfecting surfaces in the household than usual, disinfecting or wiping down groceries, disinfecting or wiping down mail or packages, stocking up food and supplies, avoiding or canceling domestic travel, avoiding or canceling international travel, not ordering takeout from restaurants, and wearing a mask when out in public).

Specifically, in Wave 2, the odds for NH-Black individuals to wash more than usual were 2.1 times greater than the odds for NH-White people when controlling for all the covariates (OR = 3.11, *p* = 0.018); the odds for NH-Black individuals to be sanitizing more than usual were 3.3 times greater than the odds for NH-White people when controlling for all the covariates (OR = 4.3, *p* =0.004); the odds for NH-Black individuals to clean the home more than usual were 2.34 times greater than the odds of NH-White people when controlling for all the covariates (OR = 3.34, *p* =0.002); the odds for NH-Black individuals to be disinfecting surfaces in the house more than usual were 4.23 times greater than NH-White people when controlling for all the covariates (OR = 5.23, *p* =0.000); the odds for NH-Black individuals to be disinfecting or wiping down groceries were 1.88 times greater than NH-White people when controlling for all the covariates (OR = 2.88, *p* = 0.003); the odds for NH-Black individuals to be disinfecting or wiping down mail or packages were 3.31 times greater than NH-White people when controlling for all the covariates (OR = 4.31, *p* = 0.000); the odds for NH-Black individuals to be stocking up food and supplies were 2.64 times greater than NH-White people when controlling for all the covariates (OR = 3.64, *p* = 0.001); the odds for NH-Black people to avoid or cancel domestic travel was 4.89 times higher than NH-White people when controlling for all the covariates (OR = 5.89, *p* = 0.000); the odds for NH-Black people to avoid or cancel international travel was 2.14 times higher than NH-White people when controlling for all the covariates (OR = 3.14, *p* = 0.002); the odds for NH-Black people to not order takeout from restaurants was 2.34 times higher than NH-White people when controlling for all the covariates (OR = 3.34, *p* = 0.003); and the odds for NH-Black people to wear a mask when in public was 3.1 times higher than NH-White people when controlling for all the covariates (OR = 4.1, *p* = 0.004).

All the preventive behaviors in Wave 3 also show statistically significant results. The odds ratio of NH-Black individuals to wash their hands more than usual was 2.36 times greater than NH-White individuals when controlling for all the covariates (OR = 3.36, *p* = 0.002); the odds ratio of NH-Black individuals to sanitize their hands more than usual was 6.63 times greater than NH-White individuals when controlling for all the covariates (OR = 7.63, *p* = 0.000); the odds ratio of NH-Black individuals cleaning their homes more than usual was 5.17 times greater than NH-White individuals when controlling for all the covariates (OR = 6.17, *p* = 0.000); the odds ratio of NH-Black individuals disinfecting their surfaces in their houses more than usual was 3.95 times greater than NH-White individuals when controlling for all the covariates (OR = 4.95, *p* = 0.000); the odds ratio of NH-Black individuals disinfecting or wiping down their groceries was 8.5 times greater than NH-White individuals when controlling for all the covariates (OR = 9.46, *p* = 0.000); the odds ratio of NH-Black individuals disinfecting their houses or wiping down their mail or packages was 9.7 times greater than NH-White individuals when controlling for all the covariates (OR = 10.69, *p* = 0.000); the odds ratio for NH-Black individuals stocking up their food and supplies was 4.22 times higher than NH-White people when controlling for all the covariates (OR = 5.22, *p* = 0.000); the odds for NH-Black people to be avoiding or canceling domestic travel was 4.55 times higher than NH-White people when controlling for all the covariates (OR = 6.55, *p* = 0.000); the odds for NH-Black people to be avoiding or canceling international travel was 4.82 times higher than NH-White people when controlling for all the covariates (OR = 5.82, *p* = 0.000); the odds for NH-Black people to not order takeout from restaurants was 3.18 times higher than NH-White people when controlling for all the covariates (OR = 4.18, *p* = 0.001); and the odds for NH-Black people to be wearing a mask when in public was 3.26 times higher than NH-White people when controlling for all the covariates (OR = 4.26, *p* = 0.000).

The results also revealed some statistically significant covariates, which are worth noting. For example, in Wave 1, results indicated that individuals with an annual household income lower than USD 20,000 had lower odds of disinfecting or wiping down groceries (OR = 0.46, *p* = 0.004) compared to individuals whose family income was greater than USD 20,000. This result is consistent with findings from other research showing that lower-income individuals might be less likely to take preventive behaviors during the COVID-19 pandemic [21]. Results of Wave 1 also showed that individuals with a higher level of Fear of COVID had slightly higher odds of disinfecting or wiping down groceries (OR = 1.06, *p* = 0.034) and stocking up food (OR = 1.08, *p* = 0.017). In addition, vaccinated individuals had much higher odds of wearing a mask than those who had not been vaccinated against COVID-19 in Wave 1 (OR = 3.22, *p* = 0.017). These results are not surprising, given that prior research has shown that people who are more worried about contracting the virus (i.e., scored higher on the FCV-19S) are more likely to practice preventive behaviors. In Wave 2 and Wave 3, having health insurance, a higher fear of COVID-19 score, and being vaccinated against COVID consistently predict one’s likelihood of practicing preventive behaviors.

## 4. Discussion and Conclusions

The present study examined preventive behavior uptake among residents living in underserved communities in Delaware. This study is innovative in the following ways: (1) it uses a longitudinal design to track changes in COVID-19-preventive behavior over time, and (2) participants were recruited from the most underserved communities in Delaware. These vulnerable populations are difficult to reach and often neglected in this field of research [16]. The findings from this study have important implications for future epidemics or pandemics.

Our study recruited and retained 208 hard-to-reach participants from the most underserved neighborhoods in Delaware. We followed them from March 2021 to November 2022. We found that, in the first wave of data collection (March 2021 to October 2021), participants of different racial backgrounds often used preventive behaviors to combat COVID-19. We did not find a statistically significant difference in terms of preventive behavior uptake between Black people and White people based on the Wave 1 data. However, the second and third waves of data showed that Black people were much more likely to engage in preventive behaviors against COVID-19 than were NH-White people in these underserved communities in Delaware. We also found that having health insurance was associated with preventive behaviors, especially when the second wave of data was collected. For example, people with health insurance, private or public, had higher odds of reporting more handwashing, hand sanitizer use, and disinfection of their homes, compared to people without health insurance. It is possible that people with healthcare providers are better informed on the most up-to-date preventive measures against COVID-19.

Delaware is a small Northeastern state consisting of three counties and a total population of about 1 million. Although a small state in terms of population and geographical size, Delaware has a diverse population in terms of race, ethnicity, and class. The Delaware population is a socio-demographic microcosm of the U.S. population. In Delaware, health disparities characterized by race and ethnicity remain substantial, consistent with national trends. The present results concerning Delaware’s most underserved communities are also consistent with prior reports showing that African Americans are more likely to practice preventive behaviors during the pandemic compared to White people. However, by employing a longitudinal design, our study makes some novel observations. For instance, we found that African Americans living in underserved communities were more likely to continue practicing preventive behaviors across all waves of the pandemic that we tested.

Further research may be conducted to investigate the reasons why African Americans were more likely to employ preventive behaviors in the battle against COVID-19. Previous studies have shown that believing in the benefits of preventive action and feeling confident in performing these actions were associated with a greater likelihood of actually adopting preventive behaviors [8]. Although our survey did not question the respondents’ beliefs about the benefits of using preventive behaviors, it is possible African Americans in our survey had more confidence in the abilities of these behaviors to protect them from contracting the virus. It is also possible that African Americans living in underserved communities employed more preventive behaviors because they had fewer medical resources, limited government support, and lower access to healthcare, whereas adopting preventive behaviors remained a viable option for protecting themselves from the virus regardless of outside support. In addition, as reported frequently by multiple media outlets, African Americans faced higher mortality rates related to COVID-19 throughout the pandemic. Knowledge of higher mortality rates may have incited African Americans to exercise additional caution and preventive behavior uptake to avoid the virus. Employing further survey techniques in addition to in-depth qualitative interviews, future research may explore these potential explanations for the higher observed preventive behaviors among African Americans.

## Figures and Tables

**Table 1:** Preventive practices to prevent the spread of COVID-19.

Preventive Practices
Avoid/Cancel Travel
Domestic
International
Use of Personal Protective Equipment (PPE)
Use of face masks
Other Infection Control Practices
Handwashing with soap and water
Use of alcohol-based hand sanitizer gel
Disinfecting or wiping down of groceries
Increased cleaning at home
Disinfecting or wiping down of mail or packages prior to use
Avoiding placing orders via takeout from restaurant

**Table 2:** Descriptive statistics.

	First Wave	Second Wave	Third Wave
Survey start date	4 March 2021	16 September 2021	31 March 2022
Survey end date	13 October 2021	25 May 2022	11 November 2022
Female (%)	58.17		
Race (%)			
NH-White	47.6		
NH-Black	36.06		
Others	16.35		
Total	100		
Less than high school degree (%)	19.71		
Family income lower than USD 20 k (%)	34.51	37.00	33.51
Health insurance Coverage (%)			
Private	31.25	26.44	24.52
Public	57.21	60.58	59.13
No insurance	11.54	12.98	16.35
Total	100.00	100.00	100.00
Fear of COVID scale	15.94 (SD = 6.48)	15.25 (SD = 6.71)	13.14 (SD = 6.03)
COVID Vaccine (%)			
Yes	58.65	63.94	69.71
COVID related behavior (%)			
More handwashing than usual	91.75	79.70	72.50
More hand sanitizer than usual	91.22	78.11	70.15
More cleaning in your home than usual	81.37	64.82	58.21
More disinfecting surfaces in your household than usual	89.76	71.07	63.18
Disinfecting or wiping down groceries	68.97	50.99	44.00
Disinfecting or wiping down mail or packages	59.61	41.50	38.31
Stocking up food and supplies	79.41	60.50	53.00
Avoiding or canceling domestic travel	72.28	57.00	42.29
Avoiding or canceling international travel	67.98	59.30	42.50
Not ordering takeout from restaurants	45.27	31.82	26.63
Wearing a mask when out in public	90.05	78.00	56.92
N	208	208	208

**Table 3:** Descriptive statistics by race and wave.

	First Wave			Second Wave			Third Wave		
	White	Black	Others	White	Black	Others	White	Black	Others
Female (%)	60.20	50.67	72.73						
No high school degree (%)	15.15	25.33	20.59						
Family Income lower than USD 20 k (%)	25.25	46.67	35.29	24.24	50.67	35.29	21.21	44.00	35.29
Health Insurance Coverage (%)									
Private	37.37	25.33	26.47	34.34	21.33	14.71	29.29	21.33	17.65
Public	56.57	64.00	44.12	56.57	68	55.88	59.6	68	55.88
No insurance	6.06	10.67	29.41	9.09	10.67	29.41	11.11	10.67	26.47
Total	100	100	100	100	100	100	100	100	100
Fear of COVID scale	15.13	17.11	15.96	13.05	17.61	16.85	12.03	14.31	13.82
	(SD = 5.97)	(SD = 7.38)	(SD = 5.67)	(SD = 5.87)	(SD = 7.50)	(SD = 4.70)	(SD = 5.62)	(SD = 6.42)	(SD = 5.97)
COVID Vaccine (%)									
Yes	70.71	44.00	55.88	70.71	56.00	61.76	71.72	65.33	73.53
COVID related behavior (%)									
More handwashing than usual	89.9	93.33	88.24	71.72	88.00	70.59	58.59	81.33	76.47
More hand sanitizer than usual	91.92	90.67	82.35	69.7	89.33	61.76	51.52	88.00	70.59
More cleaning in your home than usual	75.76	86.67	76.47	51.52	78.67	55.88	37.37	78.67	61.76
More disinfecting surfaces in your household	84.85	93.33	88.24	55.56	85.33	61.76	45.45	80.00	64.71
Disinfecting or wiping down groceries	60.61	72.00	76.47	36.36	66.67	50.00	18.18	69.33	52.94
Disinfecting or wiping down mail or packages	49.49	69.33	58.82	23.23	60.00	44.12	12.12	62.67	52.94
Stocking up food and supplies	71.72	88.00	73.53	47.47	76.00	50.00	33.33	72.00	55.88
Avoiding or canceling domestic travel	66.67	73.33	73.53	37.37	77.33	55.88	21.21	60.00	55.88
Avoiding or canceling international travel	58.59	76.00	67.65	45.45	72.33	52.94	23.4	62.67	47.06
Not ordering takeout from restaurants	32.32	54.67	52.98	17.17	44.00	38.24	12.12	40.00	32.35
Wearing a mask when out in public	87.88	88.00	82.35	68.69	88.00	64.71	38.38	72.00	55.88
N	99	75	34	99	75	34	99	75	34

**Table 4:** Logistic regression predicting the odds ratio of COVID-19-preventive behaviors in Wave 1.

	More Hand Washing Than Usual			More Hand Sanitizer Than Usual			More Cleaning in Your Home Than Usual			More Disinfecting Surfaces in Your Household Than Usual		
Predictors	OR	95% CI	*p*	OR	95% CI	*p*	OR	95% CI	*p*	OR	95% CI	*p*
White	REF			REF			REF			REF		
Black	1.73	0.52–5.74	0.371	0.84	0.27–2.66	0.771	2.02	0.87–4.71	0.103	2.61	0.86–7.97	0.092
Others	0.91	0.23–3.58	0.896	0.38	0.11–1.34	0.134	1.06	0.39–2.85	0.913	1.63	0.43–6.14	0.471
Male	REF			REF			REF			REF		
Female	1.34	0.49–3.70	0.567	1.34	0.49–3.68	0.571	1.24	0.60–2.56	0.557	1.68	0.68–4.15	0.258
Lower than HS	0.38	0.11–1.29	0.119	0.31	0.09–1.23	0.076	1.35	0.47–3.85	0.569	0.86	0.26–2.86	0.808
HS and higher	REF			REF			REF			REF		
Family income lower than 20 k	1.70	0.51–5.61	0.385	2.27	0.65–7.86	0.197	1.08	0.46–2.53	0.859	1.00	0.34–2.92	0.994
Family income 20 k and higher	REF			REF			REF			REF		
Private health insurance	0.99	0.18–5.35	0.992	0.44	0.08–2.29	0.326	1.18	0.32–4.35	0.800	1.69	0.37–7.82	0.502
Public health insurance	1.24	0.28–5.44	0.771	1.43	0.31–6.45	0.645	1.44	0.44–4.68	0.549	2.29	0.58–8.99	0.237
No health insurance	REF			REF			REF			REF		
Fear of COVID Scale	1.07	0.99–1.17	0.105	1.04	0.96–1.13	0.333	1.06	0.99–1.13	0.071	1.06	0.98–1.15	0.127
COVID vaccine	2.35	0.78–7.03	0.127	2.23	0.78–6.39	0.135	1.39	0.63–3.04	0.412	1.38	0.51–3.71	0.529
Intercept	1.56	0.19–12.41	0.676	4.31	0.53–35.22	0.173	0.69	0.14–3.47	0.655	0.76	0.11–5.18	0.781
	**Disinfecting or wiping down groceries**			**Disinfecting or wiping down mail or packages**			**Stocking up food and supplies**			**Avoiding or canceling domestic travel**		
**Predictors**	**OR**	**95% CI**	***p***	**OR**	**95% CI**	***p***	**OR**	**95% CI**	***p***	**OR**	**95% CI**	***p***
White	REF			REF			REF			REF		
Black	1.74	0.87–3.49	0.117	2.58	1.32–5.06	**0.006**	2.92	1.23–6.94	**0.015**	1.76	0.85–3.62	0.127
Others	2.26	0.85–5.97	0.101	1.76	0.75–4.12	0.195	1.08	0.41–2.86	0.871	1.7	0.64–4.50	0.283
Male	REF			REF			REF			REF		
Female	1.26	0.67–2.35	0.478	1.08	0.60–1.97	0.790	1.45	0.71–2.95	0.309	1.73	0.91–3.27	0.095
Lower than HS	0.66	0.28–1.54	0.333	1.17	0.52–2.66	0.700	0.84	0.32–2.26	0.735	0.90	0.39–2.10	0.807
HS and higher	REF			REF			REF			REF		
Family income lower than 20 k	0.46	0.22–0.97	**0.040**	0.61	0.30–1.22	0.616	0.66	0.29–1.52	0.334	0.52	0.25–1.10	0.087
Family income 20 k and higher	REF			REF			REF			REF		
Private health insurance	0.74	0.23–2.37	0.618	1.48	0.49–4.44	0.489	0.91	0.25–3.32	0.889	0.97	0.31–3.11	0.965
Public health insurance	1.34	0.47–3.79	0.580	2.06	0.77–5.52	0.149	1.31	0.41–4.18	0.464	1.84	0.66–5.16	0.247
No health insurance	REF			REF			REF			REF		
Fear of COVID Scale	1.06	1.00–1.11	**0.034**	1.02	0.97–1.07	0.389	1.08	1.01–1.15	**0.017**	1.02	0.97–1.08	0.365
COVID vaccine	0.87	0.43–1.73	0.687	1.17	0.61–2.24	0.638	1.08	0.49–2.36	0.851	1.63	0.81–3.26	0.170
Intercept	0.91	0.20–3.34	0.770	0.39	0.10–1.49	0.167	0.67	0.14–3.32	0.626	0.64	0.15–2.69	0.544
	**Avoiding or canceling international travel**			**Not ordering takeout from restaurants**			**Wearing a mask when out in public**					
**Predictors**	**OR**	**95% CI**	***p***	**OR**	**95% CI**	***p***	**OR**	**95% CI**	***p***			
White	REF			REF			REF					
Black	2.66	1.29–5.47	**0.008**	2.33	1.21–4.47	**0.011**	1.17	0.43–3.18	0.760			
Others	2.04	0.83–5.04	0.122	2.51	1.08–5.82	**0.032**	0.86	0.26–2.83	0.804			
Male	REF			REF			REF					
Female	0.64	0.34–1.22	0.177	1.16	0.64–2.12	0.622	0.87	0.35–2.12	0.754			
Lower than HS	0.61	0.27–1.40	0.244	1.52	0.69–3.37	0.302	0.41	0.14–1.19	0.101			
HS and higher	REF			REF			REF					
Family income lower than 20 k	0.78	0.38–1.61	0.507	1.04	0.53–2.04	0.918	2.42	0.85–6.90	0.098			
Family income 20 k and higher	REF			REF			REF					
Private health insurance	1.88	0.60–5.90	0.277	1.45	0.48–4.42	0.513	1.91	0.46–7.89	0.373			
Public health insurance	2.03	0.74–5.58	0.171	1.98	0.74–5.32	0.175	2.19	0.64–7.44	0.210			
No health insurance	REF			REF			REF					
Fear of COVID Scale	1.03	0.98–1.08	0.279	0.99	0.94–1.04	0.660	1.05	0.97–1.12	0.203			
COVID vaccine	1.44	0.73–2.86	0.293	0.74	0.39–1.41	0.360	3.22	1.23–8.44	**0.017**			
Intercept	0.57	0.14–2.31	0.433	0.34	0.09–1.32	0.119	0.99	0.17–5.83	0.987			

Note: *p* ≤ 0.05 are bold-faced.

**Table 5:** Logistic regression predicting the odds ratio of COVID-19-preventive behaviors in Wave 2.

	More Hand Washing Than Usual			More Hand Sanitizer Than Usual			More Cleaning in Your Home Than Usual			More Disinfecting Surfaces in Your Household Than Usual		
Predictors	OR	95% CI	*p*	OR	95% CI	*p*	OR	95% CI	*p*	OR	95% CI	*p*
White	REF			REF			REF			REF		
Black	3.11	1.21–7.99	**0.018**	4.30	1.60–11.56	**0.004**	3.34	1.54–7.25	**0.002**	5.23	2.21–12.37	**0.000**
Others	1.32	0.48–3.66	0.589	0.93	0.36–2.44	0.885	1.35	0.54–3.36	0.524	1.73	0.69–4.35	0.241
Male	REF			REF			REF			REF		
Female	1.35	0.64–2.85	0.428	1.51	0.72–3.17	0.281	1.93	0.93–3.57	0.079	1.46	0.74–2.90	0.273
Lower than HS	1.98	0.63–6.18	0.241	1.69	0.57–5.07	0.347	2.77	1.01–7.57	**0.047**	1.22	0.46–3.25	0.690
HS and higher	REF			REF			REF			REF		
Family income lower than 20 k	1.72	0.71–4.18	0.230	2.19	0.90–5.36	0.085	2.89	1.33–6.30	**0.008**	2.44	1.08–5.53	**0.033**
Family income 20 k and higher	REF			REF			REF			REF		
Private health insurance	5.01	1.55–16.13	**0.007**	4.96	1.53–16.09	**0.008**	4.52	1.43–14.29	**0.010**	3.68	1.19–11.40	**0.024**
Public health insurance	3.91	1.43–10.67	**0.008**	3.75	1.37–10.29	**0.010**	1.98	0.72–5.44	0.186	3.45	1.28–9.33	**0.014**
No health insurance	REF			REF			REF			REF		
Fear of COVID Scale	1.01	0.95–1.07	0.818	0.99	0.94–1.06	0.868	1.02	0.96–1.07	0.575	0.99	0.94–1.05	0.744
COVID vaccine	2.16	1.00–4.66	0.051	2.41	1.11–5.22	**0.025**	1.55	0.76–3.13	0.226	1.67	0.82–3.42	0.161
Intercept	0.25	0.06–1.01	0.051	0.22	0.05–0.92	**0.038**	0.12	0.03–0.45	**0.002**	0.18	0.05–0.72	**0.014**
	**Disinfecting or wiping down groceries**			**Disinfecting or wiping down mail or packages**			**Stocking up food and supplies**			**Avoiding or canceling domestic** **travel**		
**Predictors**	**OR**	**95% CI**	***p***	**OR**	**95% CI**	***p***	**OR**	**95% CI**	***p***	**OR**	**95% CI**	***p***
White	REF			REF			REF			REF		
Black	2.88	1.43–5.80	**0.003**	4.31	2.11–8.74	**0.000**	3.64	1.73–7.64	**0.001**	5.89	2.72–12.76	**0.000**
Others	1.49	0.61–3.62	0.377	2.02	0.92–5.29	0.077	1.17	0.50–2.72	0.722	2.43	0.99–6.01	0.054
Male	REF			REF			REF			REF		
Female	1.28	0.68–2.42	0.450	1.20	0.63–2.28	0.572	1.29	0.69–2.41	0.431	1.15	0.59–2.23	0.677
Lower than HS	2.54	1.02–6.31	**0.044**	1.35	0.57–3.21	0.492	1.08	0.45–2.57	0.862	1.1	0.44–2.77	0.834
HS and higher	REF			REF			REF			REF		
Family income lower than 20 k	1.98	0.96–4.09	0.065	1.16	0.56–2.40	0.681	1.46	0.70–3.03	0.310	1.51	0.70–3.27	0.293
Family income 20 k and higher	REF			REF			REF			REF		
Private health insurance	0.87	0.29–2.60	0.800	0.85	0.28–2.56	0.778	1.56	0.53–4.50	0.406	1.62	0.52–5.02	0.406
Public health insurance	1.25	0.48–3.27	0.625	0.98	0.38–2.53	0.959	1.57	0.62–3.95	0.342	3.87	1.44–10.43	**0.007**
No health insurance	REF			REF			REF			REF		
Fear of COVID Scale	1.00	0.95–1.05	0.940	1.02	0.97–1.07	0.482	0.98	0.94–1.04	0.54	1.00	0.94–1.05	0.875
COVID vaccine	1.96	0.99–3.90	0.054	1.38	0.70–2.69	0.352	1.66	0.86–3.20	0.129	2.04	1.01–4.14	**0.048**
Intercept	0.21	0.06–0.77	**0.018**	0.17	0.05–0.64	**0.008**	0.43	0.12–1.48	0.18	0.12	0.03–0.48	**0.003**
	**Avoiding or canceling international travel**			**Not ordering takeout from restaurants**			**Wearing a mask when out in public**					
**Predictors**	**OR**	**95% CI**	***p***	**OR**	**95% CI**	***p***	**OR**	**95% CI**	***p***			
White	REF			REF			REF					
Black	3.14	1.52–6.48	**0.002**	3.34	1.52–7.32	**0.003**	4.10	1.56–10.78	**0.004**			
Others	1.36	0.57–3.27	0.492	2.49	0.95–6.48	0.063	0.97	0.36–2.63	0.959			
Male	REF			REF			REF					
Female	1.07	0.57–2.00	0.837	1.39	0.69–2.80	0.364	2.13	1.01–4.50	**0.046**			
Lower than HS	1.06	0.44–2.54	0.898	2.24	0.93–5.39	0.074	1.40	0.48–4.11	0.535			
HS and higher	REF			REF			REF					
Family income lower than 20 k	0.76	0.36–1.59	0.468	1.56	0.73–3.34	0.250	1.89	0.77–4.62	0.164			
Family income 20 k and higher	REF			REF			REF					
Private health insurance	1.34	0.46–3.88	0.590	0.49	0.14–1.66	0.251	2.16	0.69–6.81	0.188			
Public health insurance	3.17	1.24–8.15	**0.016**	0.75	0.27–2.03	0.565	3.17	1.15–8.71	**0.025**			
No health insurance	REF			REF			REF					
Fear of COVID Scale	1.02	0.96–1.07	0.561	1.02	0.97–1.08	0.451	1.00	0.94–1.06	0.921			
COVID vaccine	1.47	0.76–2.83	0.255	1.20	0.58–2.46	0.622	3.21	1.49–6.92	**0.003**			
Intercept	0.27	0.07–0.94	**0.040**	0.11	0.03–0.46	**0.002**	0.22	0.05–0.90	**0.035**			

Note: *p* ≤ 0.05 are bold-faced.

**Table 6:** Logistic regression predicting the odds ratio of COVID-19-preventive behaviors in Wave 3.

	More Hand Washing Than Usual			More Hand Sanitizer Than Usual			More Cleaning in Your Home Than Usual			More Disinfecting Surfaces in Your Household Than Usual		
Predictors	OR	95% CI	*p*	OR	95% CI	*p*	OR	95% CI	*p*	OR	95% CI	*p*
White	REF			REF			REF			REF		
Black	3.36	1.55–7.26	**0.002**	7.63	3.21–18.14	**0.000**	6.17	2.95–12.91	**0.000**	4.95	2.34–10.45	**0.000**
Others	2.61	0.97–7.02	0.057	2.55	1.01–6.40	**0.047**	2.57	1.1–6.06	**0.03**	2.10	0.89–4.97	0.090
Male	REF			REF			REF			REF		
Female	1.13	0.58–2.19	0.728	0.63	0.32–1.27	0.198	1.89	0.98–3.66	0.058	1.72	0.90–3.28	0.100
Lower than HS	0.64	0.27–1.49	0.298	0.61	0.25–1.47	0.268	1.42	0.62–3.25	0.406	0.90	0.40–2.05	0.808
HS and higher	REF			REF			REF			REF		
Family income lower than 20 k	1.01	0.45–2.23	0.987	1.18	0.52–2.68	0.688	2.14	1.00–4.55	**0.049**	2.09	0.97–4.48	0.059
Family income 20 k and higher	REF			REF			REF			REF		
Private health insurance	1.76	0.61–5.06	0.294	2.16	0.70–6.65	0.181	2.87	0.97–8.45	0.056	2.68	0.92–7.79	0.070
Public health insurance	3.47	1.36–8.85	**0.009**	2.62	0.97–7.03	0.056	1.45	0.57–3.69	0.434	1.74	0.69–4.36	0.237
No health insurance	REF			REF			REF			REF		
Fear of COVID Scale	1.07	1.00–1.13	**0.037**	1.08	1.01–1.14	**0.024**	1.03	0.98–1.09	0.267	1.04	0.98–1.10	0.229
COVID vaccine	1.71	0.81–3.63	0.161	2.1	0.96–4.62	0.064	1.31	0.62–2.75	0.484	1.49	0.71–3.14	0.288
Intercept	0.18	0.50–0.67	**0.010**	0.16	0.04–0.61	**0.007**	0.10	0.03–0.39	**0.001**	0.14	0.04–0.49	**0.002**
	**Disinfecting or wiping down groceries**			**Disinfecting or wiping down mail or packages**			**Stocking up food and supplies**			**Avoiding or canceling domestic travel**		
**Predictors**	**OR**	**95% CI**	***p***	**OR**	**95% CI**	***p***	**OR**	**95% CI**	***p***	**OR**	**95% CI**	***p***
White	REF			REF			REF			REF		
Black	9.46	4.52–19.77	**0.000**	10.69	4.86–23.52	**0.000**	5.22	2.61–10.45	**0.000**	5.55	2.75–11.22	**0.000**
Others	5.47	2.23–13.40	**0.000**	7.69	2.98–19.87	**0.000**	2.56	1.11–5.91	**0.028**	5.12	2.12–12.39	**0.000**
Male	REF			REF			REF			REF		
Female	0.82	0.41–1.62	0.567	1.06	0.52–2.17	0.864	1.37	0.73–2.57	0.331	0.78	0.41–1.50	0.461
Lower than HS	1.68	0.72–3.91	0.233	1.76	0.74–4.20	0.200	1.36	0.61–3.02	0.452	1.49	0.66–3.34	0.333
HS and higher	REF			REF			REF			REF		
Family income lower than 20 k	1.38	0.64–2.96	0.413	2.03	0.92–4.47	0.079	1.58	0.76–3.28	0.218	1.19	0.56–2.51	0.650
Family income 20 k and higher	REF			REF			REF			REF		
Private health insurance	2.60	0.83–8.13	0.101	1.58	0.49–5.06	0.442	1.93	0.67–5.51	0.221	1.59	0.52–4.84	0.413
Public health insurance	2.01	0.76–5.33	0.161	0.98	0.37–2.62	0.969	2.05	0.82–5.08	0.123	2.12	0.83–5.44	0.118
No health insurance	REF			REF			REF			REF		
Fear of COVID Scale	1.05	0.99–1.11	0.118	1.04	0.98–1.10	0.247	1.01	0.95–1.06	0.843	1.02	0.97–1.08	0.395
COVID vaccine	0.95	0.44–2.05	0.892	1.28	0.58–2.84	0.542	1.38	0.67–2.84	0.389	1.79	0.84–3.81	0.132
Intercept	0.06	0.01–0.25	**0.000**	0.05	0.01–0.21	**0.000**	0.14	0.04–0.50	**0.002**	0.07	0.02–0.29	**0.000**
	**Avoiding or canceling international travel**			**Not ordering takeout from restaurants**			**Wearing a mask when out in public**					
**Predictors**	**OR**	**95% CI**	***p***	**OR**	**95% CI**	***p***	**OR**	**95% CI**	***p***			
White	REF			REF			REF					
Black	5.82	2.90–11.68	**0.000**	4.18	1.86–9.40	**0.001**	4.26	2.09–8.67	**0.000**			
Others	3.38	1.42–8.02	**0.006**	2.7	0.99–7.33	0.052	1.82	0.77–4.33	0.173			
Male	REF			REF			REF					
Female	0.81	0.42–1.54	0.515	1.22	0.58–2.55	0.597	1.58	0.83–3.01	0.162			
Lower than HS	1.06	0.47–2.38	0.885	1.73	0.74–4.04	0.202	1.45	0.63–3.33	0.377			
HS and higher	REF			REF			REF					
Family income lower than 20 k	1.33	0.63–2.78	0.451	2.33	1.04–5.24	**0.040**	1.57	0.74–3.37	0.242			
Family income 20 k and higher	REF			REF			REF					
Private health insurance	1.93	0.64–5.82	0.244	0.43	0.12–1.53	0.192	1.51	0.51–4.47	0.459			
Public health insurance	2.39	0.93–6.15	0.070	0.7	0.26–1.90	0.487	1.95	0.76–4.96	0.163			
No health insurance	REF			REF			REF					
Fear of COVID Scale	1.02	0.97–1.08	0.447	1.02	0.96–1.09	0.488	1.06	1.00–1.12	**0.045**			
COVID vaccine	1.34	0.64–2.81	0.439	2.96	1.23–7.09	**0.015**	3.21	1.49–6.92	**0.003**			
Intercept	0.09	0.02–0.34	**0.000**	0.04	0.01–0.21	**0.000**	0.05	0.01–0.19	**0.000**			

Note: *p* ≤ 0.05 are bold-faced.

## Data Availability

The data presented in this study are available on request from the corresponding author due to privacy reasons.
